# Novel Point and Combo-Mutations in the Genome of Hepatitis B Virus-Genotype D: Characterization and Impact on Liver Disease Progression to Hepatocellular Carcinoma

**DOI:** 10.1371/journal.pone.0110012

**Published:** 2014-10-15

**Authors:** Somenath Datta, Alip Ghosh, Debanjali Dasgupta, Amit Ghosh, Shrabasti Roychoudhury, Gaurav Roy, Soumyojit Das, Kausik Das, Subash Gupta, Keya Basu, Analabha Basu, Simanti Datta, Abhijit Chowdhury, Soma Banerjee

**Affiliations:** 1 Centre for Liver Research, Institute of Post Graduate Medical Education & Research, Kolkata, India; 2 Molecular Virology Laboratory, Department of Biotechnology, Jamia Millia Islamia, New Delhi, India; 3 Centre for Liver & Biliary Surgery, Indraprastha Apollo Hospital, New Delhi, India; 4 National Institute of Biomedical Genomics, Kalyani, India; Saint Louis University, United States of America

## Abstract

**Background:**

The contribution of chronic hepatitis B virus (HBV) infection in the pathogenesis of hepatocellular carcinoma (HCC) through progressive stages of liver fibrosis is exacerbated by the acquisition of naturally occurring mutations in its genome. This study has investigated the prevalence of single and combo mutations in the genome of HBV-genotype D from treatment naïve Indian patients of progressive liver disease stages and assessed their impact on the disease progression to HCC.

**Methods:**

The mutation profile was determined from the sequence analysis of the full-length HBV genome and compared with the reference HBV sequences. SPSS 16.0 and R software were used to delineate their statistical significance in predicting HCC occurrence.

**Results:**

Age was identified as associated risk factor for HCC development in chronic hepatitis B (CHB) patients (p≤0.01). Beyond the classical mutations in basal core promoter (BCP) (A1762T/G1764A) and precore (G1862T), persistence of progressively accumulated mutations in enhancer-I, surface, HBx and core were showed significant association to liver disease progression. BCP_T1753C, core_T147C, surface_L213I had contributed significantly in the disease progression to HCC (p<0.05) in HBeAg positive patients whereas precore_T1858C, core_I116L, core_P130Q and preS1_S98T in HBeAg negative patients. Furthermore, the effect of individual mutation was magnified by the combination with A1762T/G1764A in HCC pathogenesis. Multivariate risk analysis had confirmed that core_P130Q [OR 20.71, 95% CI (1.64–261.77), p = 0.019] in B cell epitope and core_T147C [OR 14.58, 95% CI (1.17–181.76), p = 0.037] in CTL epitope were two independent predictors of HCC in HBeAg positive and negative patients respectively.

**Conclusions:**

Thus distinct pattern of mutations distributed across the entire HBV genome may be useful in predicting HCC in high-risk CHB patients and pattern of mutational combinations may exert greater impact on HCC risk prediction more accurately than point mutations and hence these predictors may support the existing surveillance strategies in proper management of the patients.

## Introduction

Chronic infection with hepatitis B virus (HBV) is the commonest cause of hepatocellular carcinoma (HCC) which is fast emerging as a leading cause of cancer related morbidity and mortality globally [1]–[3]. Decades of successful infection with repetitive cycles of injury and necroinflammation followed by regeneration and repair are the characteristic of chronic hepatitis B (CHB). HCC is the final outcome of CHB and liver fibrosis with or without cirrhosis is the hallmark of progressive CHB. These various clinical outcomes of CHB as a result of complex host–virus immune interplay have a consequential relationship and cirrhosis nearly always precedes HCC development in CHB infection [4]. It is important to note that HBV has tremendous abilities of immune evasion [5] by either acquiring mutations at viral immune dominant epitopes [6], [7] or several critically important sites essential for progressive CHB related to liver diseases [8], [9]. More intriguingly, it has been demonstrated that viral mutations are accumulated over a period of time and such accumulation of mutations in the viral genome might have pathogenic relevance [9], [10].

Viral genetic factors such as genotype and classical mutations at basal core promoter (BCP) and precore (PC) have been shown to correlate with clinical outcomes in HBV related liver diseases [11], [12]. Such associations have interesting geographical distribution that has clinical and evolutionary significance. Apart from the classical BCP and PC mutations, several mutations in surface, core, HBx and regulatory regions have been described to occur in CHB over time that might help in defining clinical situations–if studied sequentially [13]–[15]. India, with an intermediate endemicity of chronic HBV infection [16] and predominant chronic infection with HBV-genotype D [17] presents a unique HCC scenario. Firstly, while the burden of HBV associated cirrhosis is fairly high, the reported prevalence of HCC is relatively low [18], [19], although HBV is the most common cause of HCC in India [20]. Secondly, detection of HCC in India is fairly late and hence, the poor prognosis – mandating the need for in depth studies into different aspects of HBV associated HCC in India. Studying the pattern of viral mutations in specific morphological–clinical category of hepatitis B virus infection might provide insights that not only help in elucidating the pathogenesis but also predicting HCC occurrence.

We report here the progressive accumulation of naturally occurring viral mutations in a group of treatment naïve/ineligible HBV infected subjects with different histological subgroups of fibrosis and HCC to seek evidence for any discernible and predictable viral genetic variations that occur in HCC.

## Materials and Methods

### Ethics statement

Institutional ethical review committee (Institutional Ethics Committee, I.P.G.M.E & R, 244, A.J.C Bose Road, Kolkata-20, India) has approved the study (Inst./IEC/646). Also informed written consent was obtained from each patient who was willing to participate in the study.

### Study subjects

A total of 92 Subjects enrolled for detailed virological assessment in current study between the periods of June 2009 to December 2012 form two distinct groups:

- Those presenting with different clinical manifestations of CHB for evaluation to clinical Hepatology services of the School of Digestive and Liver Diseases, Institute of Post Graduate Medical Education and Research, Kolkata (n = 80).
- Those undergoing orthotropic liver transplantation for HBV related liver diseases at the centre for liver and biliary diseases, Indraprastha Apollo Hospitals, New Delhi (n = 12).

Detailed history, clinical, biochemical, radiological evaluations and virological examinations were performed on each patient as clinically indicated.

Patients were included into the study only if: (a) treatment naïve/ineligible with chronic HBV infection (b) age above 18 year (c) absence of other co-infections i.e. negative for anti-HCV and anti-HIV (d) no history of alcohol use (e) willingness to comply with study protocol.

### Evaluation of liver disease status

For the purpose of categorization of the included patients according to the status of liver diseases focussing on grade of liver fibrosis (LF), presence of liver cirrhosis (LC) and HCC, each patient underwent liver stiffness measurement (LSM) with Fibroscan that uses Transient elastographic technique. LSM less than 7 kPa was taken as normal and more than 12.5 kPa was taken as cut-off for the diagnosis of cirrhosis [21], [22]. Liver biopsy was done in non-cirrhotic patients who provided consent for the procedure and the histological status of the explanted liver was used in cases who underwent liver transplantation. All subjects were subsequently sub-classified into four categories: (1) ‘no liver fibrosis’ (nLF) (n = 22) was diagnosed either by absence of fibrosis in histology or by LSM value less than 7 kPa. (2) ‘liver fibrosis less than cirrhosis’ (LF) (n = 15) was defined histologically and supplemented with LSM value between 7.1 and 12.5 kPa and endoscopic confirmation of absence of varices and imaging feature of absence of portal hypertension, (3) ‘liver cirrhosis’ (LC) (n = 23) was diagnosed either by presence of histological feature of cirrhosis or by clinical, endoscopic, radiological parameters and LSM evidences in persons who did not undergo liver biopsy and (4) hepatocellular carcinoma (HCC) (n = 32) was diagnosed as per standard protocol using ultrasound, spiral/triphasic dynamic CT and serum Alfa-fetoprotein (AFP) values, apart from histology of explants. Blood samples were collected from all patients and the separated sera were preserved in −80°C till further use.

### Serological parameters tests

Each serum sample was subjected to blood biochemistry such as total protein, albumin, globulin, bilirubin, prothrombin time (INR), ALT/SGPT, AST/SGOT using commercially available kits from Bayer Diagnostics, India. Serological markers such as HBsAg, HBeAg, anti-HBe and anti-HCV were verified using commercially available ELISA kits from General Biologicals, Taiwan and Biomerieux, Boxtel, Netherlands. The normal ranges for AST and ALT are 2–45 IU/L and 2–40 IU/L respectively.

### HBV Viral DNA isolation and Quantification

HBV DNA was extracted from serum using QIAamp DNA Mini Kit (Qiagen Inc., Valencia, CA) following manufacturer's instructions. Real-Time PCR was used to quantify viral DNA (Applied Biosystems, Foster city, USA). The lower limit of detection is 250 copies/ml.

### Amplification of full-length HBV genome and DNA Sequencing

Full length HBV genome (∼3.2 kb) was amplified by one-step PCR method using P1/P2 primers [23] and for samples with low viral load, nested PCR was performed using MP1/R5 and F3/MP2 primers (Table 1). The QIA Quick Gel Extraction Kit (Qiagen, CA) was used to purify PCR products and the sequences were determined using the Big Dye terminator v3.1 cycle sequencing kit (ABI, USA) on an automated DNA Sequencer (ABI Prism 3130). Editing and analysis of the DNA sequences were done using Seqscape V2.5 (ABI, USA) software.

**Table 1:** List of primers used to generate PCR product from HBV genome.

Primer Name	Primer Sequences	Nucleotide Position
P1^a^	5 -CCGGAAAGCTTGAGCTCTTCTTTTTCACCTCTGCCTAATCA- 3′	1821–1841
P2^a^	5′-CGGAAAGCTTGAGCTCTTCAAAAAGTTGCATGGTGCTGG- 3′	1806–1825
MP1^b^^,^ c	5′-GAGCTCTTCTTTTTCACCTCTGCCTAATCA- 3′	1821–1841
MP2^b^	5′-GAGCTCTTCAAAAAGTTGCATGGTGCTGG- 3′	1806–1825
2412F^c^	5′-GCGTCGCAGAAGATCTCAATC-3′	2412–2432
R4^c^	5′ -AGAGGACAAACGGGCAACA- 3′	462–480
R5^b^	5′ -AAAGCCCAAAAGACCCACAAT-3′	997–1017
F3^b^^,^ c	5′ -CGCCTCATTTTGTGGGTCAC- 3′	2801–2820
R3^c^	5′ -AACTGGAGCCACCAGCAG-3′	57–74
F9^c^	5′ -TACCACAAGAGCACATTATACA- 3′	908–929
1482F^c^	5′-CTACCGTCCCCTTCTTCATC-3′	1482–1501

a Primers used for one-step full-length PCR,

b Primers used for nested PCR,

c Primers used for sequencing.

### Phylogenetic Tree construction

To construct a Phylogenetic tree, 92 full-length HBV DNA sequences obtained from this study were compared with representative 55 complete HBV sequences of eight different genotypes (A–H) retrieved from GenBank using Clustal W (MEGA 4.1) software. To confirm the reliability of the phylogenetic tree analysis, bootstrap re-sampling and reconstruction were carried out 5000 times. Sequence variability was analysed with the help of multiple sequence alignment.

### Accession numbers of submitted Nucleotide sequences

The full genome nucleotide sequences of ninety-two HBV isolates used for analysis in this paper are available in Genbank database http://www.ncbi.nlm.nih.gov/GenBank/index.html by the accession numbers KC875251- KC875342.

### Statistical Analysis

All statistical analyses were performed using “R” software version 2.14.1 and SPSS version 16.0. To evaluate the association of a mutation in progression of the disease, ANOVA was performed. Two groups (Non-HCC and HCC) were compared using Mann-Whitney U tests for continuous variables and the χ2 test or Fisher's exact test for categorical variables. Multivariable analysis applying the method of binary logistic regression was performed to find out the independent risk factors for HCC taking the clinical parameters and HBV genetic mutations, which were found to be significant in univariate analysis as independent variables. Two-tailed p values of <0.05 was considered as statistically significant.

## Results

92 treatment naïve patients with chronic HBV infection were screened in the present study at inclusion. The amplified full-length genome or overlapping fragments were sequenced directly and analysed. The phylogenetic tree analysis with complete HBV sequences (n = 92) (Figure S1) showed the presence of HBV-genotype D (n = 68; 74%), HBV-genotype C (n = 14; 15%) and HBV-genotype A (n = 10; 11%). Since we were interested in HBV genotype D, subsequent mutation analysis was pursued exclusively in the sixty-eight HBV-genotype D isolates. During extracting data, we observed escalating trend in several clinical parameters (data not shown) and also in viral mutation rate accompanying with the progression of the disease stages but to identify the risk factor of HCC occurrence, we categorized the four disease stages nLF, LF, LC and HCC into two groups such as HCC and non-HCC which includes nLF, LF and LC.

### Demographic profile of the study subjects with chronic HBV-genotype D infection

Table 2 shows the demographic, clinical and biochemical details of sixty-eight patients. 84% (57/68) of the subjects were males and 32% (22/68) had HCC. HCC patients were significantly older than non-HCC group (mean age, 52.8±10.9 yrs. in HCC vs. 38.2±15.2 yrs. in non-HCC; p = 0.0001) and 73% (16/22) of the HCC had underlying cirrhosis. However, neither HBV DNA load (5.02±1.41 and 5.0±1.84 log copies/ml in HCC and non-HCC, respectively; p = 0.97) nor the frequency of HBeAg positivity [40.9% (9/22) vs.45.5% (20/46) in HCC vs. non-HCC; p = 0.79)] was differed significantly between the HCC and non-HCC subjects (Table 2).

**Table 2:** Clinical, biochemical and demographic profile of sixty-eight patients included in the study.

Parameters	All Patients (n = 68)	Patients without HCC (n = 46)	Patients with HCC (n = 22)	p value
**Men; n (%)**	57 (84)	37 (80)	20 (91)	NS
**Age [year]^Δ^,**	42.9±15.5	38.2±15.2	52.8±10.9	0.0001
**HBeAg Positivity; n (%)**	29 [43.9]	20 [45.5]	9 [40.9]	0.79
**Anti-HBeAg Positivity; n (%)**	34 [53.1]	21 [48.8]	13 [61.9]	0.42
**HBV DNA [log_7_ Copies/ml]^ Δ^**	5.01±1.7	5.0±1.84	5.02±1.41	0.97
**Prevalence of cirrhosis; n (%)**	36 [52.9]	20 [43.5]	16 [72.7]	0.01
**Total Bilirubin, [mg/dL] ^β^,**	1.5 [0.4–33.3]	1.3 [0.4–20]	2.5 [0.5–33.3]	0.03
**ALT [IU/L]^β^**	65 [19–744]	56 [19–744]	90 [54–254]	0.004
**AST [IU/L]^ β^**	86 [22–440]	55 [22–408]	123 [70–440]	0.001
**ALP [IU/L]^ β^**	158 [55–702]	136 [55–702]	298 [59–580]	0.005
**INR^Δ^**	1.39±0.42	1.36±0.43	1.44±0.42	0.52
**Albumin [g/dL]^ Δ^**	3.7±0.94	3.8±0.96	3.4±0.84	0.07
**Globulin [g/dL]^ β^**	3.3 [0.8–6.3]	3.2 [0.8–6.3]	3.8 [1.2–4.8]	0.09

SD =  Standard deviation; NS =  Not significant; ALT =  Alanine aminotransferase; AST = Aspartate aminotransferase; INR =  International normalized ratio; ALP = Alkaline phosphatase; IU =  International unit; Δ Parameters presented as Mean±SD; β parameters presented as Median [Range]; δ Mutations with statistically significant difference between the groups are presented.

### Pattern of distribution of mutations across the HBV genome among different stages of liver diseases

The prevalence of mutations, which were showing an increasing trend from nLF (no liver fibrosis) to HCC through LF (liver fibrosis) and LC (liver cirrhosis) is summarized in Table 3. Only those mutations were considered for analysis, which were noted with mutation frequency more than 6% (i.e., present in at least ≥4 samples) among 68 HBV-genotype D subjects.

**Table 3:** Frequencies of mutations showing escalating trend with the progression of the liver diseases from no liver fibrosis (nLF) or liver fibrosis (LF) to liver cirrhosis (LC) and hepatocellular carcinoma (HCC).

Position of mutations in HBV genome	non-HCC			HCC n [%] n = 22	*p-*value [One way Anova] upto LC	*p-*value [One way Anova] upto HCC
Regulatory regions (Nucleotide change)	nLF n[%] n = 17	LF n[%] n = 9	LC n[%] n = 20			
**Enhancer I/HBx promoter (1021–1373)**						
T1050G/A	3 [17.65]	2 [22.22]	7 [35.00]	12 [54.55]	0.17	0.035
A1053G	1 [5.88]	1 [11.11]	10 [50.00]	11 50.00]	0.26	0.081
T1050G/A+A1053G	0 [0.00]	1 [11.11]	5 [25.00]	7 [31.82]	0.04	0.008
**Enhancer II (1636–1741)**						
C1653T	0 [0.00]	2 [22.22]	5 [25.00]	7 [31.82]	0.26	0.079
**BCP (1751–1769)**						
T1753C	1[5.88]	2 [22.22]	7 [35.00]	7 [31.82]	0.12	0.14
A1762T/G1764A	6 [35.29]	4 [44.44]	13 [65.00]	17 [77.27]	0.13	0.01
**Precore (1814–1900)**						
G1862T	0 [0.00]	0 [0.00]	2 [10.00]	4 [18.18]	0.33	0.05
T1858C	1 [5.88]	1 [11.11]	3 [15.00]	8 [36.36]	0.054	0.08
G1896A	0 [0.00]	0 [0.00]	0 [0.00]	6 [27.27]	-	0.225
**ORFs**(Amino acid change)						
**Pre S1**						
S98T (T3139A)	0 [0.00]	1 [11.11]	4 [20.00]	7 [31.82]	0.041	0.001
**Surface**						
T125M (C528T)	9 [52.94]	7 [77.78]	11 [55.00]	7 [31.82]	0.952	0.408
T127L (C534T)	0 [0.00]	0 [0.00]	4 [20.00]	9 [40.91]	0.333	0.057
L213I (T791A)	0 [0.00]	0 [0.00]	9 [45.00]	11 [50.00]	0.333	0.085
**HBx**						
H94Y (C1653T)	0 [0.00]	2 [22.22]	5 [25.00]	7 [31.82]	0.269	0.079
I127T (T1753C)	1 [5.88]	2 [22.22]	7 [35.00]	7 [31.82]	0.129	0.106
K130M (A1762T)/V131I (G1764A)	6 [35.29]	4 [44.44]	13 [65.00]	17 [77.27]	0.13	0.01
**Core**						
T12S (A1934T)	3 [17.65]	3 [33.33]	7 [35.00]	11 [50.00]	0.278	0.036
S35T (T2003A)	0 [0.00]	1 [11.11]	3 [15.00]	6 [27.27]	0.173	0.017
T67N (C2100A)	4 [23.53]	3 [33.33]	7 [35.00]	9 [40.91]	0.247	0.037
E113Q (G2237C)	0 [0.00]	3 [33.33]	5 [25.00]	8 [36.36]	0.212	0.212
I116L (A2246C)	3 [17.65]	4 [44.44]	8 [40.00]	11 [50.00]	0.157	0.157
T147C (A2339T/C2340G)	0 [0.00]	1 [11.11]	5 [25.00]	9 [40.91]	0.041	0.003
P130Q (C2289A)	0 [0.00]	0 [0.00]	3 [15.00]	7 [31.82]	0.333	0.059
**Polymerase**						
L12V (A2339T/C2340G)	0 [0.00]	1 [11.11]	5 [25.00]	9 [40.91]	0.041	0.003
F221Y (T791A)	0 [0.00]	0 [0.00]	9 [45.00]	11 [50.00]	0.333	0.085

Significant mutations were indicated in bold (p≤0.05) and marginal significant values were represented in italics.

### *i. Mutations in the regulatory regions of HBV:*

Out of two enhancers and four promoters in the HBV genome, five mutations were noted with rising prevalence through progressive disease stages of nLF-LF-LC and HCC. Two point mutations in enhancer-I region (nt. 1021–1234), T1050G/A and A1053G were noticed first time in HBV-genotype D. Although the prevalence of A1053G was extremely high in both LC (50%) and HCC (50%), it was only 6% in nLF and 11% in LF (p = 0.081) whereas, T1050G/A and their combination T1050G/A+A1053G showed significant escalating trend over the course of infection from nLF (18%, 0%) to HCC (54%, 32%) through LF (22%, 11%) and LC (35%, 25%) (p = 0.035, 0.008 respectively). The most common mutation in BCP (nt.1751–1769) A1762T and G1764A, which reduce the expression of HBeAg were always detected in combination with extremely high frequency in each of the disease stages, 35% in nLF, 44% in LF, 65% in LC and 77% in HCC (p = 0.01). Mutation C1653T in enhancer-II and T1753C in BCP, which were reported previously as risk factor for HCC in HBV-genotype C [24], were not observed with significantly enhancing frequencies from nLF to HCC through LF and LC in HBV-genotype D infection (p = 0.079, 0.14 respectively).

### *ii. Mutations in four ORF regions:*

#### *Surface ORF*

The HBV envelope composed of three forms of Surface antigens: large (encoded by preS1/pre S2/S), middle (coded by pre S2/S) and small (Surface gene). The most notable amino acid substitution observed in overlapping B and T cell epitopes of pre S1 region was S98T, which showed significant association with disease progression from LF (11%) to HCC (32%) through LC (20%) (p = 0.001) and was undetected in nLF. In pre S2 and pre S1 regions, few scattered deletions were observed but were not significantly linked to HCC. Interestingly, two mutations in “a determinant region” of surface T125M and T127L were noted inversely correlated with each other. T125M was found with high frequency (>52%) in nLF, LF and LC but low in HCC (32%) whereas T127L was noted only in LC (20%) and HCC (41%). Additionally, a single point mutation L213I in C-terminal region of surface was encountered at a high frequency in LC (45%) and HCC (50%) patients but was statistically insignificant (p = 0.085).

#### *HBx ORF*

No new amino acid substitution in B cell (aa. 29–48) or T cell (aa. 91–105) immune epitopes of HBx ORF [24] was significantly altered with progression of CHB to HCC, except the changes in BCP region, which induce mutations in overlapping HBx ORF such as T1753C to I127T, A1762T and G1764A to K130M and V131I.

#### *Precore/core ORF*

The occurrence of precore mutation, T1858C was noticed with an increasing frequencies of 5.88% in nLF, 11% in LF, 15% in LC and 36% in HCC (p = 0.08). Other two precore mutations G1862T and G1896A, which were frequently found in HBeAg negative patients, were noted exclusively in the advanced stages such as LC and HCC (10%, 0% and 18%, 27%; p = 0.05 and 0.225 respectively) (Table 3).

Variabilities in T helper (T_h_) epitopes (aa. 35–45 and 48–69), B cell epitopes (aa.76–89, 105–116, 130–135) and Cytotoxic T lymphocyte (CTL) epitopes (aa.18–27, 50–69, 74–83, 141–151) of core protein were found important in disease progression to LC and HCC [25]. Overall three mutations in T_h_ epitope (T12S, S35T, T67N) and one in CTL epitope (T147C) of core showed significant association with HCC (p<0.05) (Table 3). Few other mutations E113Q (36%), I116L (50%) and P130Q (32%) in B cell epitope were observed with high frequency in HCC but the differences were insignificant.

#### *Polymerase ORF*

The preS/S ORF is completely overlapped to the polymerase gene. The L213I mutation (TTA to ATG) in surface leads to F221Y and A222T dual mutations in reverse transcriptase (RT) domain of HBV polymerase resulting a change in polymerase activity or viral replication [25] whereas the mutation S98T in pre S1 (L12V in Polymerase) lies in the non-essential spacer region of the polymerase. No other mutations were noticed in the RT domain of the polymerase gene.

### Identification of point mutations associated with HCC

**Figure 1:**
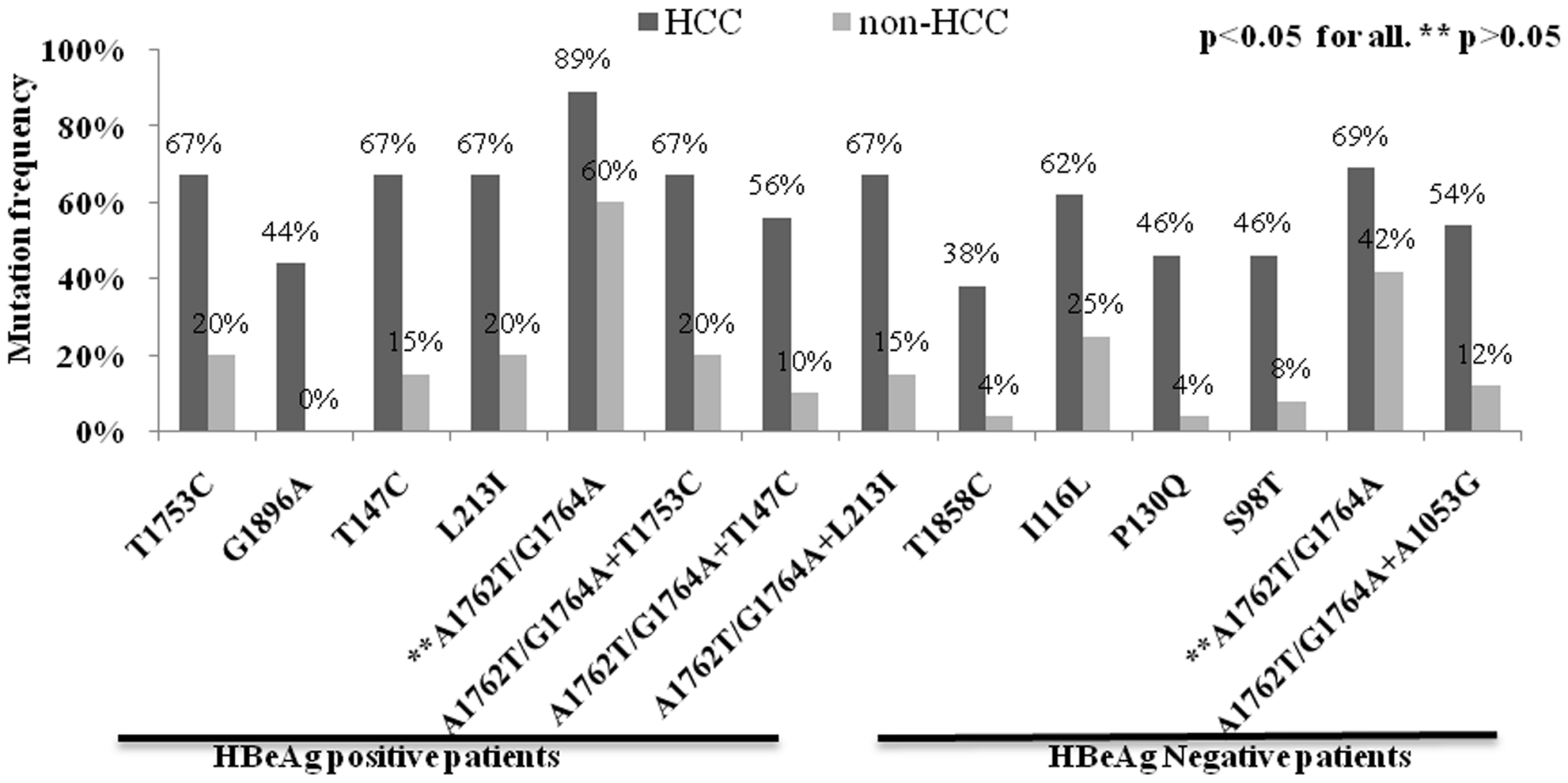
Frequencies of naturally occurring single and combination of mutations accumulated in the HBV genome were found significantly higher in hepatocellular carcinoma (HCC) than non-HCC patients. p<0.05 was considered as significant. ** indicates p>0.05.

The significance of mutations in relation to HCC occurrence was further investigated by univariate logistic regression analysis between two groups' non-HCC (nLF+LF+LC) and HCC. As shown in Figure 1, age was an associated risk factor for HCC development (p<0.01) but the common A1762T/G1764A dual mutation in BCP failed to show significant correlation in both HBeAg positive [89% (8/9)] and negative HCC [69% (9/13)] compared to non-HCC [60% (12/20); p = 0.2 and 42% (10/24); p = 0.17 in HBeAg positive and negative cases respectively]. Patients harbouring T1753C in BCP, T147C in CTL epitope of core and L213I mutation in surface were found more in HBeAg positive HCC than non-HCC cases (67% vs. 20%; 67% vs. 15% and 67% vs. 20% between HCC and non-HCC respectively). The odds ratios (OR) of these three mutations for HCC risk were significant at 7.3 [95% CI (1.037–67.9), p = 0.032] for both T1753C and L213I and 10.1 [95% CI (1.334 106.5), p = 0.01] for T147C (Table S1). While the prevalence of T1858C in precore, two mutations in B cell epitopes of core I116L and P130Q and S98T in pre S1 were predominant in HBeAg negative HCC (38% vs. 4%; 62% vs. 25%, 46% vs. 4% and 46% vs. 8% in HCC vs. non-HCC respectively) with significant ORs for HCC risk at 13.2 [95% CI (1.227–705.1), p = 0.014], 4.6 [95% CI (0.913–26.2), p = 0.039], 17.9[95% CI (1.747–938.7), p = 0.004] and 8.7 [95% CI (1.221–107.9), p = 0.013] respectively.

### Identification of combination of mutations associated with HCC

To evaluate any synergistic effect of HBV mutations on HCC development, the differences in frequencies of mutational combinations were determined using a pattern analysis program in “R” to generate all possible combinations. From a total of more than 100 combination patterns, four patterns (3 in HBeAg positive and 1 in HBeAg negative) were identified which were linked to HCC development. The prevalence of those combinations were significantly more in HCC than non-HCC, such as A1762T/G1764A with T1753C in enhancer II [67% (6/9) vs. 20% (4/20), p = 0.03], A1762T/G1764A with T147C in core region [56% (5/9) vs. 10% (2/20), p = 0.016)] and A1762T/G1764A with L213I in surface region [67% (6/9) vs. 15% (3/20), p = 0.01)] found in HBeAg positive patients whereas A1762T/G1764A with A1053G in enhancer-I region [54% (7/13) vs. 13% (3/24), p = 0.016] was important in HBeAg negative HCC patients (Figure 1, Table S1). The ORs of all four combinations for HCC prediction were also significant (Table S1).

### Multiple logistic regression analysis to determine the risk factors for HCC development

Finally, to identify the independent predictive factor for the development of HCC multiple logistic regression analysis was performed. Older age and the presence of T147C [OR 14.58, 95% CI (1.17–181.76), p = 0.037] in CTL epitope and P130Q [OR 20.71, 95% CI (1.64–261.77), p = 0.019] on B-cell epitope were identified as independent predictor of HCC in HBeAg positive and negative patients respectively (Table 4). No combination of mutations was found as independent risk factor, which might be due to small number of study subjects.

**Table 4:** Multivariate analysis to identify independent risk factors for HCC development in HBeAg positive and negative patients.

Mutations	Multiple logistic regression analysis	
In HBeAg Positive	OR [95% CI]	p value [Fisher's Exact]
T1753C	11.48 [0.95–139.09]	0.055
T147C	14.58 [1.17–181.76]	**0.037**
L213I	5.61 [0.59–53.05]	0.133
**In HBeAg negative**		
T1858C	3.08 [0.19–50.44]	0.43
I116L	2.53 [0.37–17.49]	0.348
P130Q	20.71 [1.64–261.77]	**0.019**
S98T	8.91 [0.98–81.08]	0.052

p value <0.05 was considered as significant.

## Discussion

We have demonstrated here that progressive accumulation of mutations in HBV genome exert a powerful effect on the occurrence of HCC in HBV infected subjects and such influence of individual mutations are magnified by mutational combinations, indicating synergism rather than an additive effect which is similar to the data suggested from recent meta-analysis studies [26]–[29]. This is the first study dealing with the full-length genome of HBV and observed mutations in the entire genome of HBV are important for progression of liver diseases. Although, two Indian studies by the same group had delineated several mutations associated with HBV-genotype D related HCC [30], [31], but detailed analysis to shape that into a defined predictive group had not been done. Thus this study put into focus several findings at the level of individual genes, beyond the well described classical ones in the precore and basal core promoter regions which are well known to be associated with progressive liver diseases–that are relatively novel and delineate their significance as candidate risk factor of HCC.

According to the previous studies in HBV genotype B, genotype C [32]–[34] and few studies with genotype D [30], [31], [35], [36], BCP_A1762T/G1764A, precore_G1896A, EnhII_C1653T, BCP_T1753V mutations and pre S2 deletions are frequently associated risk factors of HCC. Our study had also found a significant association of BCP_A1762T/G1764A mutation to the liver disease progression from nLF to HCC (Table 3). This mutation is associated with high viral replication through the removal of nuclear receptor-binding factor and generation of new binding site for hepatocyte nuclear factor (HNF) [37], which is responsible for HCC occurrence and also recurrence after resection [38],[39]. Although this dual mutation was highly prevalent in both HBeAg positive and negative HCC (88.88% and 69.23% respectively) compared to non-HCC (60% and 42% respectively), no significant correlation was noted with disease manifestations in binary logistic regression analysis (*P*>0.05, Table S1), which might be because of small study subjects. This combination was also not found associated with HCC in previous study with genotype D [30], [31] but showed correlation in HCC with HBV-genotype C [33], [26]. The frequency of BCP_C1653T mutation in the enhancer-II region was increasing with disease severity (Table 3) but it did not correlate well with LC or HCC, which was previously documented as an independent risk factor for HCC with HBV-genotype C [40] suggesting its genotype specific role in HCC development.

In a previous study from India BCP_T1753C mutation was reported as risk factor for LC rather than HCC [30], [31] which was similar to our analysis from frequency distribution (Table 3) but univariate analysis further identified this mutation as risk factor for HCC in HBeAg positive patients. This mutation was also reported as risk factor for HBV-genotype D related HCC from Mongolia [35]. Again, in consistence with the previous report in HBV genotype C, this mutation in combination with BCP_A1762T/G1764A showed significant association with HBeAg positive HCC development (Figure 1). Recently it has been found that this combination of mutations exhibited an enhanced viral replication phenotype like BCP_A1762T/G1764A alone [36] but its exact role in hepatocarcinogenesis is unclear.

The role of HBV enhancer-I region/HBx promoter in HCC has been studied very infrequently, except in one study [41] and almost never in HBV genotype D. However, we observed several sequence heterogeneities in this region. Previously, the G1053A mutation at the p53 factor like binding site was shown to enhance the expression of HBx and it was correlated well with the high replication efficiency of the HBV-genotype C virus [41]. But the clinical relevance of the high prevalence of A1053G and T1050G/A mutation in HBV-genotype D and its association with HCC is not clear (Table 3). These mutations might not affect HBsAg but could alter polymerase activity as another mutation in this region, T1055A has been reported to affect the conformation of two helices and impair HBV DNA-primer interaction [42]. Few new combination of mutations in the enhancer-I region were also noted in LC and HCC such as T1029C/A1032G (>30%), T1059A/A1105C (>25%), and C1249T/T1250C (>15%) indicating their importance in LC and HCC development (data not shown) but not found significant in this study. The B cell (aa. 26–45) and T cell epitope (aa. 116–127) of HBx are frequently altered in HBV-genotype C and B [24], but no significant sequence heterogeneity was noted in the epitopes of HBV–genotype D, except three mutations I127T (T1753C), K130M (A1762T) and V131I (G1764A), though H94Y (T1653C) was present with high frequencies in both LC and HCC (Table 3) as observed in previous study [30].

HBV core region is an important target of immune mediated viral clearance by inducing CTL, T_h_ cells and B cells response [43]. Mutations in CD4^+^ T_h_ epitopes (aa 1–20, 28–47, 50–69, 72–105, 108–165) are usually associated with immune evasion, minimum immune mediated hepatitis and viral persistence than CD8^+^ CTL epitopes [44]. However association of core gene mutations and HCC has not been well delineated before. Although a large number of mutations in CTL epitopes showed significant association with cirrhosis and HCC [25], no such significant change was observed in the core antigenic CTL region (18–27 amino acids) in this study. While three mutations in T_h_ epitopes (T12S, S35T, T67N), T147C in CTL epitope and two mutations I116L, P130Q in B cell epitopes showed significant association with disease severity (Table 3). Koschel et al showed that deletion at codon 12 is defective in virion secretion [45] while other two mutations S35T, T67N might be able to evade the host immune response for their persistence at low levels [46], [47]. Interestingly, the most frequently observed naturally occurring core protein mutation found in CHB [48], [49] and also in HCC patients I97L or F97L [50], [51] which is associated with “immature virion secretion” was completely absent in Indian patients with HBV-genotype D while among the “low secretion core variants” such as P5T/A/S, a new variant P5R was noted with high frequency in HBV-genotype D related HCC in previous Indian study [30], [31]. This mutation was also not found in our study. But the compensatory mutation of codon 97 at codon 130, P130Q was found as independent risk factor for HCC in HBeAg negative patients. Mutation T147C was not documented as HCC risk factor previously with respect to any genotype. From our multivariate analysis it is obvious that the two core mutations, T147C and P130Q could be potential predictor of HCC in high-risk HBeAg positive and negative CHB patients respectively (Table 4). In addition, T147C with classical BCP mutation A1762T/G1764A was noted significantly high in HCC than non-HCC and hence these mutations in combination could predict the progression of liver diseases more precisely.

We have also studied the naturally occurring mutations in T and B-cell epitopes of pre S1 and pre S2 regions. A recent meta-analysis study has clearly showed the association of pre S deletion with the progression of liver diseases to HCC in HBV genotype C with OR 3.77 [95% CI (2.57–5.52)] [14] whereas point mutations and risk of HCC are rarely studied. To our surprise, few insignificant deletions were identified in pre S1 and pre S2 regions in HCC patients, which are in agreement with two different studies who found deletion in pre S2 might be genotype specific as observed more in HBV-genotype C than genotype B [51], [52]. One point mutation L213I observed in the overlapping surface and polymerase gene (F221Y/A222T) in combination with classical BCP mutations A1762T/G1764A showed association with HCC in HBeAg positive patient (Figure 1). This mutation might be associated with immune evasion and active viral replication. The S98T mutation found in the overlapping T and B cell epitope region of pre S1, which is the binding site of heat shock protein 70 (HSP70) (aa74–118) [53] might be an independent predictor for HCC in HBeAg negative patient (Table 4).

The clinical significance of G1896A mutation is ambiguous. In accordance with other studies this mutation was found in advanced form of liver diseases [53] while some studies showed either its occurrence in inactive carrier [54] or it has no association with clinical outcomes [14]. Another precore mutation, T1858C, which makes a pair with G1896A to stabilize the viral encapsidation signal and enhance viral replication, was observed significantly associated with more aggressive course of liver diseases in HBeAg negative HCC (Figure 1) similar to previous study [55].

In accordance to the previously identified risk factors for HCC such as male sex, age>50 yrs, high serum ALT level, HBeAg, high HBV DNA level, HBV genotype C and D and point or combination of mutations in BCP, precore and pre S as powerful contributor in CHB patients [56]–[58], this study has identified late age, high ALT and several point and combination of mutations in BCP, precore, core and surface as predictors of HCC in HBV genotype D infected CHB patients (p<0.05).

Thus in conclusion, comparing the full-length genome sequences of HBV-genotype D isolates from HCC and non-HCC groups, core gene exhibited the most significant alteration in mutation frequency (p<0.02). A comparatively larger group of each morphological – Fibrosis phenotype used in the current study might improve the strength of our observations and we are planning that. Despite this limitation in small sample numbers, these findings, depicting a robust correlation of HBV genomic mutational combinations on HCC development in HBV genotype D, are unique and are likely to have connotations as well as relevance for designing downstream studies in this area using high throughput molecular tools. In addition, analogous to the clinical utility that a pattern of combined mutation in somatic genes provide in several cancers [59], [60], viral mutational combination patterns may also prove useful in picking up high risk CHB infections at risk for developing HCC and supplement existing surveillance strategies in this group, as a supportive tool.

## Supporting Information

Figure S1 **Phylogenetic tree analysis of full-length genome of ninety-two HBV isolates from treatment naive patients with different clinical stages along with fifty-five reference sequences of eight different HBV genotypes (A–H), retrieved from Genbank including two HBV sequences of non-primate origin.** Reference sequences are indicated by the genotypes followed by accession number and origin of the countries. The tree was constructed using neighbour joining (Nj) method by MEGA 5.10. software and bootstrap re-sampling and re-construction were repeated 5000 times. ◂ and ▸ indicate no liver fibrosis (anti-clockwise) and Liver fibrosis (clockwise) while ▪ and • denote Liver cirrhosis and Hepatocellular carcinoma respectively.
(TIF)

Table S1 **Frequencies of single or combination of mutations associated with HBeAg positive and negative HCC patients.**
(DOC)
