# Supplementary figures and images for: Novel Point and Combo-Mutations in the Genome of Hepatitis B Virus-Genotype D: Characterization and Impact on Liver Disease Progression to Hepatocellular Carcinoma
Source: PLoS One. 2014 Oct 15;9(10):e110012. doi: 10.1371/journal.pone.0110012 (PMC4198185; doi:10.1371/journal.pone.0110012)

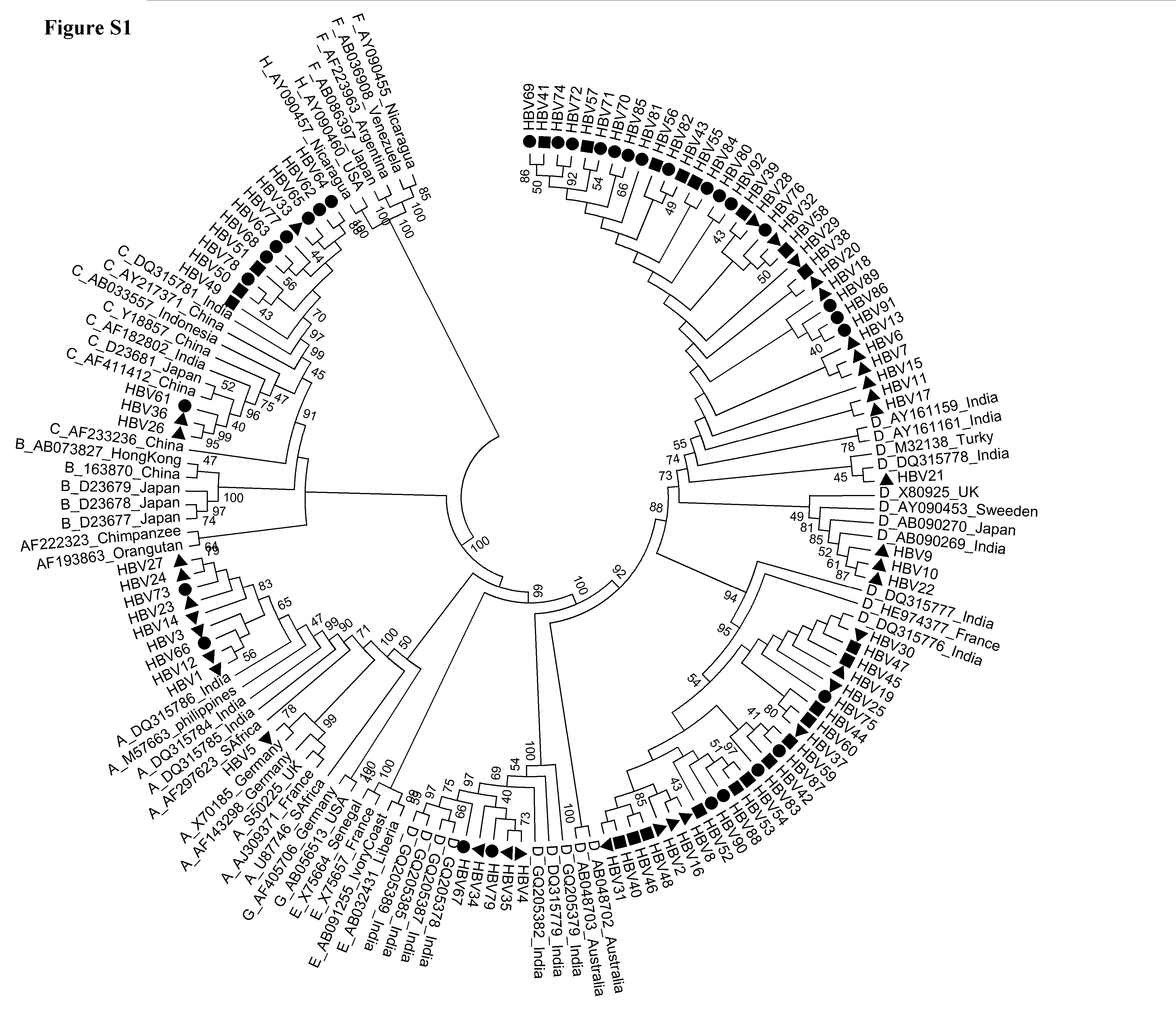

Supplement: Figure S1 — Phylogenetic tree analysis of full-length genome of ninety-two HBV isolates from treatment naive patients with different clinical stages along with fifty-five reference sequences of eight different HBV genotypes (A–H), retrieved from Genbank including two HBV sequences of non-primate origin. Reference sequences are indicated by the genotypes followed by accession number and origin of the countries. The tree was constructed using neighbour joining (Nj) method by MEGA 5.10. software and bootstrap re-sampling and re-construction were repeated 5000 times. ◂ and ▸ indicate no liver fibrosis (anti-clockwise) and Liver fibrosis (clockwise) while ▪ and • denote Liver cirrhosis and Hepatocellular carcinoma respectively. (TIF) [file pone.0110012.s001.tif]
