# Supplementary material for: Novel Point and Combo-Mutations in the Genome of Hepatitis B Virus-Genotype D: Characterization and Impact on Liver Disease Progression to Hepatocellular Carcinoma
Source: PLoS One. 2014 Oct 15;9(10):e110012. doi: 10.1371/journal.pone.0110012 (PMC4198185; doi:10.1371/journal.pone.0110012)
Supplement: Table S1 — Frequencies of single or combination of mutations associated with HBeAg positive and negative HCC patients. (DOC) [file pone.0110012.s002.doc]

**Table S1:**

| **Mutations**  **In HBeAg positive** | **non-HCC**  **n=20** | **HCC**  **n=9** | **Univariate logistic regression analysis**  **OR [95% CI]** | **p value**  **(Fishers’**  **Exact)** |
| --- | --- | --- | --- | --- |
| T1753C | 4 [20.00] | 6 [66.67] | 7.3 [1.037 - 67.9] | **0.032** |
| T147C | 3 [15.0] | 6 [66.67] | 10.1 [1.334 106.5] | **0.01** |
| L213I | 4 [20.00] | 6 [66.67] | 7.3 [1.037 - 67.9] | **0.032** |
| A1762T/G1764A | 12 [60.00] | 8 [88.88] | 5.1 [0.5-265.3] | 0.201 |
| A1762T/G1764A/T1753C | 4 [20] | 6 [66.67] | 7.3 [1.04 - 67.9] | **0.031** |
| A1762T/G1764A/T147C | 2 [10] | 5 [55.56] | 10.1 [1.16 - 142.8] | **0.016** |
| A1762T/G1764A/L213I | 3 [15] | 6 [66.67] | 10.1 [1.33 - 106.57] | **0.01** |
| Age | 34.6±17.8 | 52.5±13.4 |  | **0.01** |
| **In HBeAg negative** | **n= 24** | **n= 13** |  |  |
| T1858C | 1 [4.17] | 5 [38.46] | 13.2 [1.227 705.1] | **0.014** |
| I116L | 6 [25] | 8 [61.54] | 4.6 [0.913 - 26.2] | **0.039** |
| P130Q | 1 [4.17] | 6 [46.15] | 17.9[1.747 - 938.7] | **0.004** |
| S98T | 2 [8.33] | 6 [46.15] | 8.7 [1.221 - 107.9] | **0.013** |
| A1762T/G1764A | 10 [41.67] | 9 [69.23] | 3.1 [0.626-17.6] | 0.17 |
| A1762T/G1764A/A1053G | 3 [12.5] | 7 [53.85] | 7.6[1.27 - 60.5] | **0.016** |
| Age | 40.08±11.8 | 52.92±9.4 |  | **0.002** |

p value <0.05 indicates significant value. HBeAg status of two samples was undetermined.
